# Supplementary material for: Transcriptomic Study of Different Stages of Development in the Testis of Sheep
Source: Animals (Basel). 2024 Sep 25;14(19):2767. doi: 10.3390/ani14192767 (PMC11475124; doi:10.3390/ani14192767)
Supplement: Supplementary file 1 [file animals-14-02767-s001.zip › animals-3167687-supplementary.pdf]

Supplementary Table S1. Primer information used for RT-qPCR.

Gene Name	Primer Sequences (5′ – 3′)	Product Length (bp)	Annealing Temperature (°C)
FOXO1	F: AGATCGACCCGGACTTCGAG<br>R: GAAGGCCCATTCTGCCATAGC	178	59
RAB3B	F: CTTCCTTCCTCTTCCGCTAT<br>R: TCACTCGCTTCTCGTGGC	108	56
MARK1	F: TTCCTCCTGCTGTATCAT<br>R: ACATCTTTGTCCCACTCC	82	50.21
SOX9	F: AGCCGGACCTGAAGAAGGAGA<br>R: ACCAGCGTCCAGTCGTAGCC	97	60.19
GATA6	F: TCCCTACGCCTCGCATCCCT<br>R: GTACATCTCCTCCGGCTGCTC	202	60.46
YAP1	F: TGACGGATGGGAACAAGC<br>R: GGGTCTAGCCAAGAGGTG	84	53.92
GATA4	F: CGCCAACTGCCAGACCACCA<br>R: AGAGGCCGCAGGCATTACA	80	61.34
IGF1	F: TCAGCAGTCTTCCAA CCCAA<br>R: TGAAGGCGAGCAAGCACA	117	56.18
ITGB1	F: GCACGATGTGATGATTTA<br>R: CTTTGCTACGGTTGGTTA	118	50.33
SMAD4	F: GAACGAGTTGTATCACCTGGAA<br>R: CGATGGCTGTCCCTCAAA	114	54.84
β-actin	F: CTTCCAGCCTTCCTTCCTGG<br>R: GCCAGGGCAGTGATCTCTTT	180	57.83

Supplementary Table S2. Statistics of RNA-Seq data quality.

Sample Name	Library Number	Raw Reads (bp)	Clean Reads (bp)	Error Rate	Q20	Q30	GC pct
M0-1	1	6807823500	6725489934	0.03	95.55%	90.02%	57.76%
M0-2	2	6187174800	6095242611	0.03	94.69%	88.60%	60.19%
M0-3	3	6703239900	6568372905	0.03	92.19%	85.39%	68.72%
M3-1	4	6222604800	6068134820	0.03	91.45%	84.12%	72.87%
M3-2	5	6336157800	6204792016	0.03	93.01%	86.43%	67.35%
M3-1	6	6763242300	6649146734	0.03	93.70%	87.16%	67.56%
M6-1	7	6532348800	6444826521	0.03	95.19%	89.48%	62.92%
M6-2	8	5381160300	5305805054	0.03	95.48%	89.83%	62.17%

M6-3	9	5324697300	5236709846	0.03	94.79%	88.60%	62.43%
Y1-1	10	5877133200	5787631568	0.03	95.35%	89.40%	58.76%
Y1-2	11	6809175900	6689482036	0.03	95.09%	88.71%	62.60%
Y1-3	12	5330871000	5270157090	0.03	95.81%	90.33%	56.12%

Error rate: overall sequencing error rate for the data; Q20 and Q30: percentages of total bases with Phred values above 20 and 30, respectively; GC pct: percentage of C and G among the four bases in clean reads.

Supplementary Table S3. Statistics of reads aligned to genomic regions.

Sample Name	Total Reads	Total Map	Unique Map	Multi Map
M0-1	45097740	40163149 (89.06%)	37809972 (83.84%)	2353177 (5.22%)
M0-2	40938472	34720817 (84.81%)	32361220 (79.05%)	2359597 (5.76%)
M0-3	44276616	33131256 (74.83%)	28688795 (64.79%)	4442461 (10.03%)
M3-1	41059606	28994937 (70.62%)	23773946 (57.90%)	5220991 (12.72%)
M3-2	41882056	33453995 (79.88%)	28455373 (67.94%)	4998622 (11.93%)
M3-1	44782976	36031519 (80.46%)	31393386 (70.10%)	4638133 (10.36%)
M6-1	43260896	37683975 (87.11%)	33952547 (78.48%)	3731428 (8.63%)
M6-2	35634506	31105572 (87.29%)	28085264 (78.81%)	3020308 (8.48%)
M6-3	35220324	30377457 (86.25%)	26883639 (76.33%)	3493818 (9.92%)
Y1-1	38923952	34705060 (89.16%)	32170194 (82.65%)	2534866 (6.51%)
Y1-2	45031270	39093773 (86.81%)	34904921 (77.51%)	4188852 (9.30%)
Y1-3	35314810	32086043 (90.86%)	30263724 (85.70%)	1822319 (5.16%)

Sample: sample name; total reads: number of clean reads upon quality control; total map: number (percentage) of reads aligned to the reference genome; unique map: number (percentage) of reads aligned to a unique region of OAR3.1 (subsequently analyzed for quantitation); multi map: number (percentage) of reads with alignment to many locations of OAR3.1.

Supplementary Table S4. Statistics of reads aligned with the reference genome.

Sample Name	Exonic Region	Intronic Region	Intergenic Region
M0-1	29433310 (73.28%)	8772854 (21.84%)	1956985 (4.87%)
M0-2	25411152 (73.19%)	7593450 (21.87%)	1716215 (4.94%)
M0-3	25629779 (77.36%)	5866187 (17.71%)	1635290 (4.94%)
M3-1	20681970 (71.33%)	6309877 (21.76%)	2003090 (6.91%)
M3-2	25725559 (76.90%)	6090825 (18.21%)	1637611 (4.90%)
M3-1	29118692 (80.81%)	5310484 (14.74%)	1602343 (4.45%)
M6-1	31697724 (84.11%)	4532146 (12.03%)	1454105 (3.86%)

M6-2	25311911 (81.37%)	4406676 (14.17%)	1386985 (4.46%)
M6-3	24440870 (80.46%)	4440388 (14.62%)	1496199 (4.93%)
Y1-1	25110768 (72.35%)	7568853 (21.81%)	2025439 (5.84%)
Y1-2	30894535 (79.03%)	6231475 (15.94%)	1967763 (5.03%)
Y1-3	23229737 (72.40%)	7059498 (22.00%)	1796808 (5.60%)

Exonic, intronic and intergenic regions: numbers (percentages) of reads with alignment to the exonic, intronic and intergenic regions, respectively.
